# Polarization disorder of decidual NK cells in unexplained recurrent spontaneous abortion revealed by single-cell transcriptome analysis

**DOI:** 10.1186/s12958-022-00980-9

**Published:** 2022-07-27

**Authors:** Dingchen Pan, Qian Liu, Le Du, Yang Yang, Guojing Jiang

**Affiliations:** 1 Obstetrics and Gynaecology Department, Shuguang Hospital of Shanghai University of Traditional Chinese Medicine, 528 Zhangheng Road, Shanghai, 201203 China; 2 Shuguang Clinical College, Shanghai University of Traditional Chinese Medicine, 528 Zhangheng Road, Shanghai, 201203 China; 3 Experiment Centre for Science and Technology, Shanghai University of Traditional Chinese Medicine, 1200 Cailun Road, Shanghai, 201203 China

**Keywords:** Unexplained recurrent spontaneous abortion, Maternal–fetal interface, Single-cell RNA-seq, Decidual nature killer cell, Extracellular matrix, Immune tolerance

## Abstract

**Background:**

Unexplained recurrent spontaneous abortion (URSA) is one of the most common diseases in pregnancy and is mainly caused by immune disorders. The foetus is similar to semiallogeneic maternal tissue, so the balance of immune tolerance must be dynamically maintained during pregnancy. Decidual natural killer (dNK) cells primarily mediate the immune tolerance microenvironment at the maternal–fetal interface. By using single-cell RNA sequencing (scRNA-seq) and high-throughput transcriptome sequencing analysis, we explored the characteristic distribution of dNK cells in URSA patients.

**Methods:**

Control maternal–fetal interface tissue (from normal pregnant women, *n* = 3) and case maternal–fetal interface tissue (from patients with URSA, *n* = 3) samples were analysed by scRNA-seq and high-throughput transcriptome sequencing.

**Results:**

By scRNA-seq, we demonstrated the maturation process of the transition of dNK cells from cytotoxic characteristics to immune tolerance in transcriptome analysis. Moreover, compared with normal pregnant women, serious disturbances in the polarization process of dNK cells were found in URSA. Simultaneously, the transcriptional level of the extracellular matrix (ECM) in URSA patients showed a significant decrease. The dNK cells interacted with extravillous trophoblasts to achieve immune-tolerant polarization.

**Conclusions:**

Insufficient expression of KIRs during dNK cell differentiation might be a key reason why polarized dNK cells still had high cytotoxic reactivity in URSA patients. Abnormal expression of ECM may affect the interaction of dNK cells with EVTs, making dNK cells immature. Both resulted in maternal immune intolerance to the foetus during pregnancy.

**Supplementary Information:**

The online version contains supplementary material available at 10.1186/s12958-022-00980-9.

## Introduction

Recurrent spontaneous abortion (RSA) is the occurrence of two or more spontaneous abortions in a row, including consecutive biochemical pregnancies [1]. With the increasing number of women of childbearing age globally, the prevalence of RSA is also increasing. It has become one of the most common diseases afflicting women. According to statistical data, the incidence of RSA is approximately 1%-5% worldwide, and clinical studies have found that the miscarriage rate of the second pregnancy in such patients is as high as 70%-80% [2]. The mechanisms of RSA can be divided into two categories: nonimmune and immune. Approximately 50% of RSA is caused by immunologic factors, known as unexplained recurrent spontaneous abortion (URSA). In reproductive immunology, pregnancy is an analogue of the semiallogeneic transplantation process. Even though the foetus’s paternal antigens are directly exposed to the maternal immune system, the immunological tolerance microenvironment ensures a successful pregnancy at the decidua, also called the maternal–fetal interface. This process is essential for immune tolerance between immune cells, such as NK and T cells and extravillous trophoblasts (EVTs) in the maternal–fetal interface [3, 4].

During pregnancy, the foetus is a semiallogeneic tissue of the mother, and the maternal immune system should maintain both immune tolerance to the foetus and immune defence against infection [5, 6].The number of decidual natural killer (dNK) cells reaches more than 70% of decidual immune cells during the first trimester of pregnancy [7–9]. After a certain period, the proportion of dNK cells gradually decreases, though they still act as the most important component of the immune regulatory cells in the maternal–fetal interface. Acquisition of immune tolerance function is accompanied by adequate maturation of dNK cells, and the interaction between dNK cells and EVTs is essential for the process of polarization. Unlike most somatic cells that express classical major histocompatibility complex class I (MHC-I) molecules (HLA-A or HLA-B), invasive EVTs mainly express HLA-C and nonclassical MHC-I molecules (HLA-E and HLA-G) [10–12]. The inhibitory receptors of dNK cells, such as killer immunoglobulin-like receptors (KIRs), interact with these special MHC-I molecules, limiting the cytotoxicity of dNK cells and facilitating the maintenance of immune tolerance balance [13, 14]. However, when the intercellular crosstalk in the maternal–fetal interface is broken, inadequate maturation of dNK cells leads to downregulation of immune tolerance and upregulation of cytotoxicity [15]. Immune imbalance induces a series of pregnancy complications, such as insufficient trophoblast invasion and defective decidual vascular remodelling, which may eventually trigger URSA [16]. However, the factors that interfere with the polarization of dNK cells are diverse [17], and the dominant pathological mechanism has still not been clarified.

In recent years, some studies [18–20] using single-cell RNA-seq(scRNA-seq) technology have analysed the transcriptome regulation codes of cells in the maternal–fetal interface, revealed the distribution and developmental track of the cell subtype in the decidua, and defined the regulation of immune tolerance during the uterine spiral artery recasting process in a successful pregnancy. ScRNA-seq methods provide a viable way to systematically study pregnancy immunity. To deeply reveal the imbalance mechanism of immune tolerance regulation during URSA, we collected decidual tissues from typical URSA patients and normal pregnant women, and dNK cells were used as the main research objects. Through transcriptome analysis following scRNA-seq, the dysregulated characteristics that affected dNK cells in URSA patients during the process of polarization were explored.

## Results

### Identification the cell atlas at the decidua in scRNA-seq

To obtain maternal–fetal interface tissues from typical URSA patients, we excluded patients who had a previous autoimmune history, uterine anomalies, congenital prethrombotic state, acquired prethrombotic state, endocrine diseases, parental chromosome abnormalities or abnormal chromosomal abnormalities in previous embryos. Finally, we diagnosed these patients with URSA. We obtained 6 human maternal–fetal interface tissues during early pregnancy, of which 3 were URSA patients and 3 were normal control women. The average age of patients in the URSA group was 32.33 years old, and that in the normal pregnancy group was 31.67 years old. The average gestational age of the URSA group was 7.66 weeks, and that of the normal control group was 7.24 weeks. All 3 URSA patients had a history of embryo loss twice, and they underwent electric negative-pressure aspiration within 1 week of the diagnosis of foetal arrest (Table 1). And in URSA group, we did the fetal chromosome examination to exclude the error caused by the fetal chromosomal abnormality.

**Table 1:** The inclusion criteria of case and control group

*Inclusion Criteria*	*Case(mean* ± *SD)*	*Control(mean* ± *SD)*
*Number of previous spontaneous abortions*	2.00 ± 0.00	0.00 ± 0.00
*Age(years old)*	32.33 ± 0.58	31.67 ± 0.58
*Gestational age(weeks)*	7.66 ± 0.21	7.24 ± 0.17

**Fig. 1:**
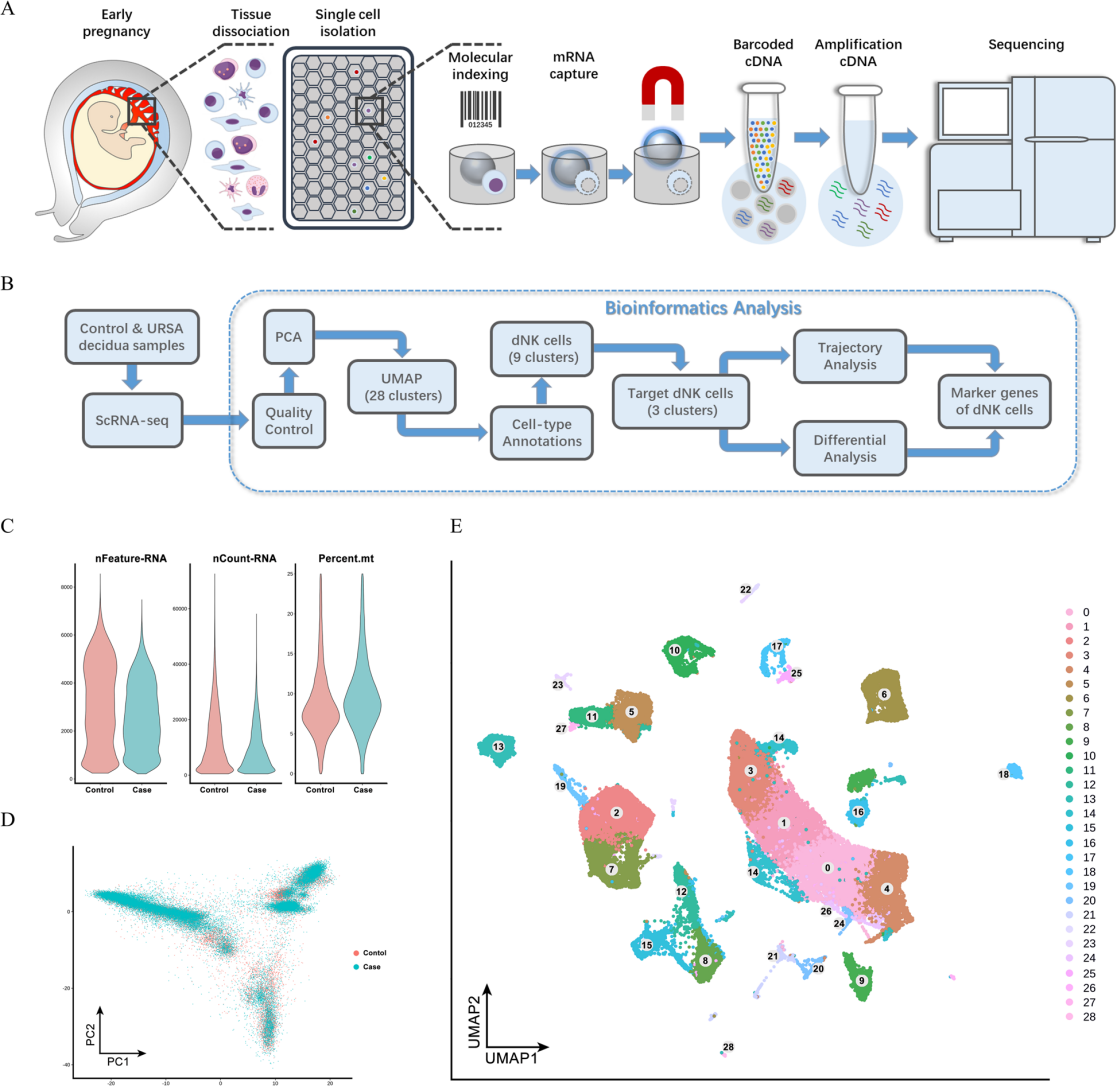
Identification the cell atlas at the decidua in scRNA-seq. **A** The maternal–fetal interface tissues were made into single-cell suspensions and then sorted and analysed using the BD Rhapsody™ system. **B** The changes in dNK cells in URSA patients were studied by transcriptome analysis. **C** A total of 63,249 high-quality cell samples were obtained for further analysis. **D** The analysis scope was preliminarily determined by principal component analysis (PCA) with related gene screening. **E** Twenty-nine clusters (0 to 28) of cells were identified by bioinformatics analysis of gene expression characteristics of all single-cell samples by UMAP visual analysis

In the study, the maternal–fetal interface tissues were made into single-cell suspensions and then sorted and analysed using the BD Rhapsody™ system (Fig. 1A). After scRNA-seq, we focused on the changes in dNK cells from URSA patients through transcriptome analysis (Fig. 1B). First, data quality control was performed by assessing the single-cell expression profile matrix, including filtering cells and genes. The cells with more than 25% mitochondrial UMI, less than 500 UMI, or 200 genes were regarded as low-quality cells and were excluded from the downstream analysis, and 63,249 high-quality cell samples were obtained for further analysis (Fig. 1C). According to the first 50 pcs, the analysis scope was preliminarily determined by principal component analysis (PCA) with related gene screening (Fig. 1D). Subsequently, Seurat was used for uniform manifold approval and projection (UMAP) visual analysis, and 29 (0 to 28) cell clusters were identified by bioinformatics analysis of the gene expression characteristics of all single-cell samples (Fig. 1E).

### Changes in proportions of immune cells in URSA decidua

**Fig. 2:**
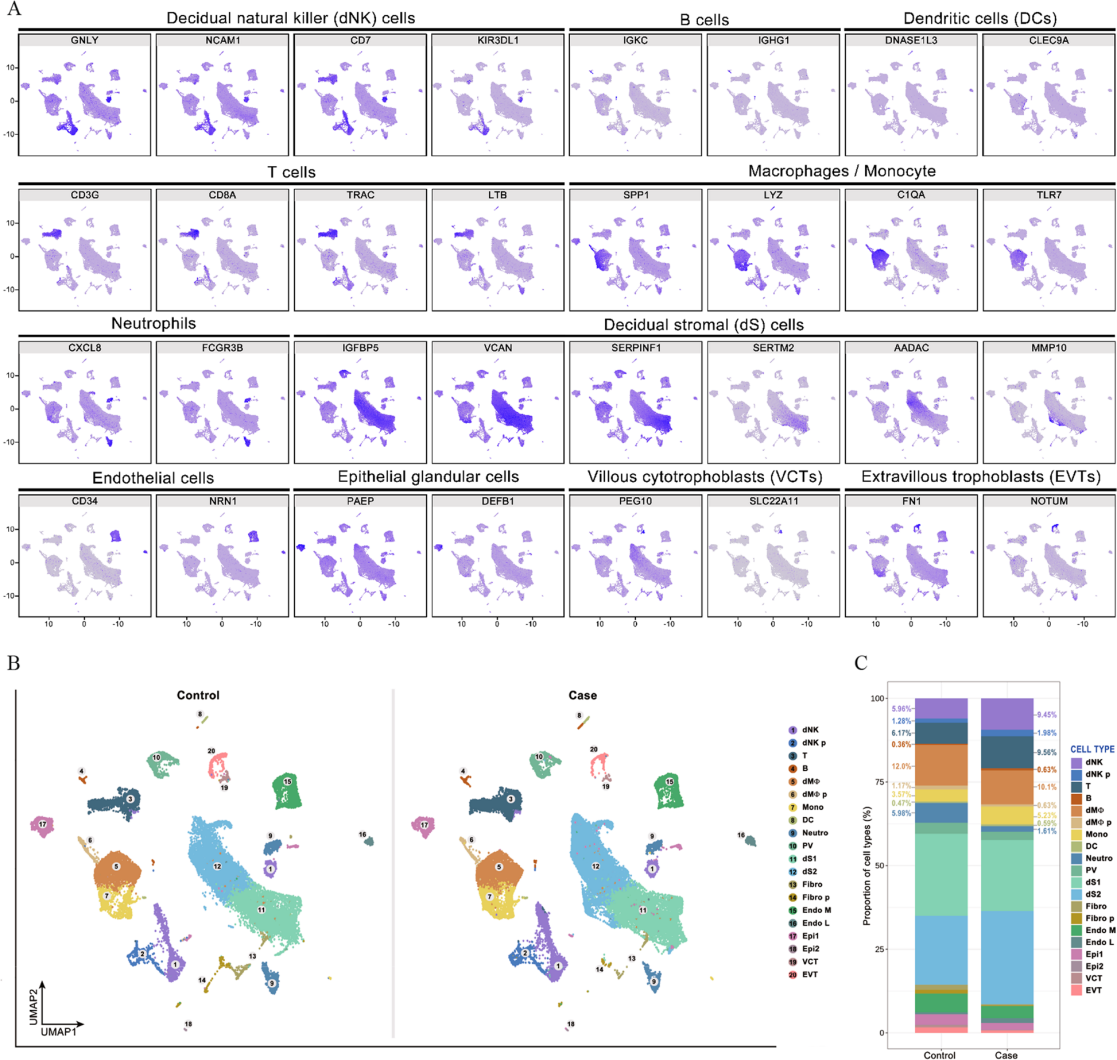
Changes in the proportions of immune cells in URSA decidua. **A** The cells were comprehensively identified as 20 cell types according to highly expressed genes. **B** The number of immune cells in URSA cell samples (case) was increased compared with that in control samples (control). **C** The proportions of NK cells and proliferating NK cells in the maternal–fetal interface of URSA patients and the control sample

Further analysis by Seurat identified a total of 31,112 marker genes (Supplement Table 1) from all cell samples. The marker genes significantly expressed by each cell cluster were subjected to gene expression difference analysis (Fig. 2A, Supplement Table 2). According to the calculation of Single R software and manual judgements, the cell types were comprehensively identified as 20 cell types, which included NK cells, T cells, B cells, dendritic cells (DCs), macrophages, monocytes, neutrophils, decidual stromal cells, endothelial cells, epithelial gland cells, villous cytotrophoblasts (VCTs), and EVTs. Among them, NK cells highly expressed *GNLY*, *NCAM1*, *CD7* and *KIR3DL1*, and T cells highly expressed *CD3G*, *CD8A*, *TRAC*, and *LTB* (Fig. 2A). Compared with the control samples, some distribution changes in URSA cell samples were clearly observed in UMAP, especially the increasing number of immune cells (Fig. 2B). The proportions of NK cells and proliferating NK cells in the maternal–fetal interface of URSA patients were 9.45% and 1.98%, respectively, while the proportions in the control sample were 5.96% and 1.28%, respectively (Fig. 2C). Similarly, the proportion of T cells in URSA patients compared with normal pregnant women also increased significantly. Due to the critical role of NK cells in maintaining immune tolerance during pregnancy, subsequent transcriptome analyses were conducted on NK cells and their polarization processes.

### Different states of dNK cells in URSA

**Fig. 3:**
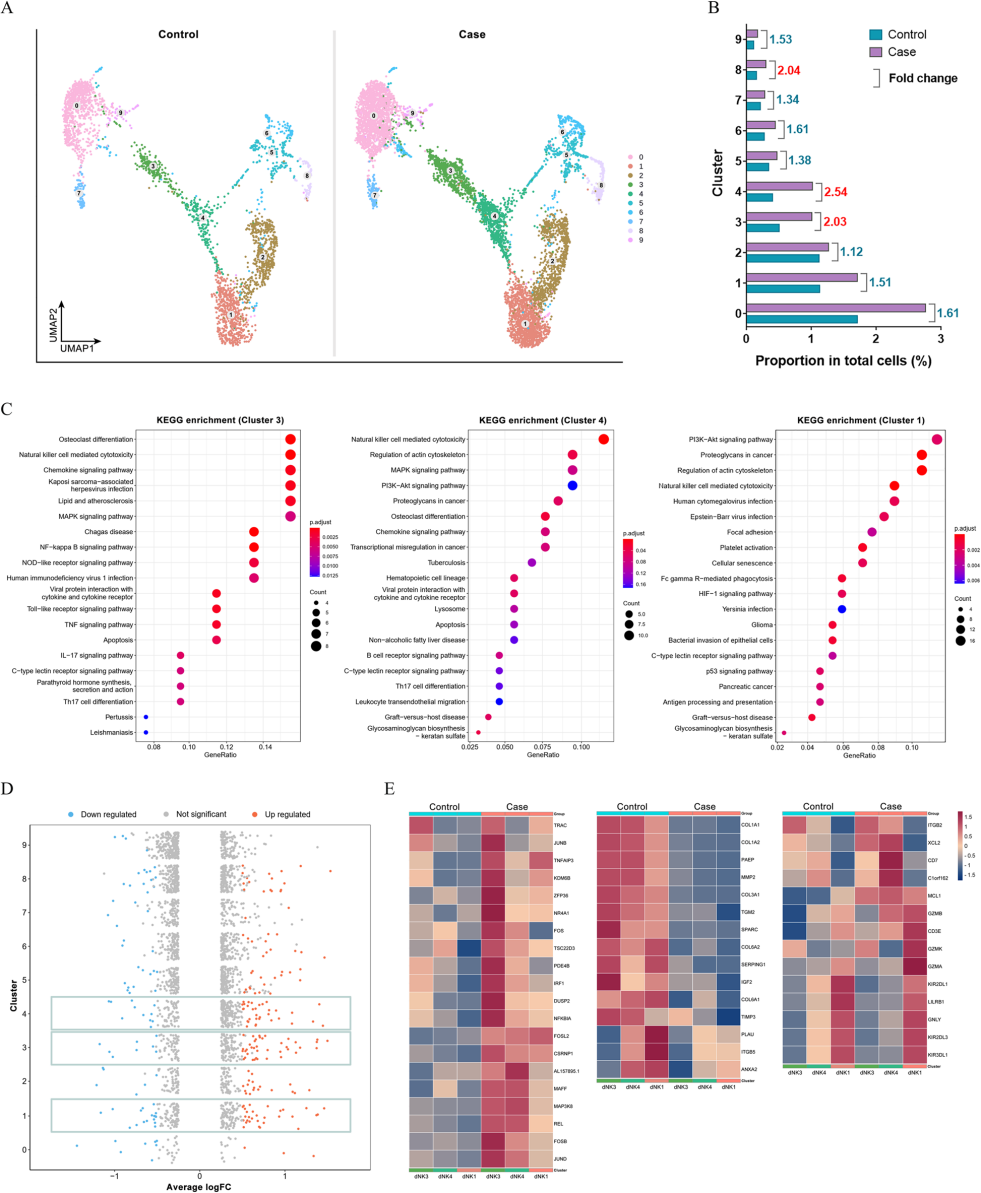
Different states of dNK cells in URSA. **A** NK cells in the decidua were divided into 10 clusters (0 to 9) of characteristic cells according to differences in transcription. **B** The proportion of each NK-cell cluster in total cells was calculated, and the changes were compared between the URSA and control groups. **C** The molecular mechanism and pathway of NK Clusters 3, 4, and 1 were defined through KEGG enrichment analysis. **D** The differences in transcriptional levels between URSA and normal pregnant women were analysed in NK Clusters 3, 4, and 1. **E** The expression levels of differentially expressed genes in Clusters 3, 4, and 1 in URSA patients and normal pregnant women were compared by heatmap

Through further clustering with UMAP, NK cells in the decidua were divided into 10 clusters (0 to 9) of characteristic cells according to differences in transcription (Fig. 3A). Comparing the differences in the distribution of URSA and control NK cells in UMAP, the degree of increase in the number of NK cells in each cluster of URSA was different. Meanwhile, the proportion of each NK-cell cluster in the total cells was calculated, and the changes were compared between the URSA and control groups. There were three groups of NK-cell subsets whose numbers increased more than twofold in the URSA sample. The fold changes of Clusters 3, 4 and 8 were 2.03, 2.54, and 2.04, respectively (Fig. 3B). Cluster 8 was similar to Clusters 5 and 6, and all of them were distinguished from other cell types due to differences of their proliferative levels. Interestingly, we found not only increased proportions of Clusters 3 and 4 in the decidua of URSA patients, but also comparable transcriptional characteristics between the two clusters of cells. Clusters 3 and 4 might be associated with the sequential differentiation of Cluster 1 because of the close spatial distribution of UMAP. Therefore, the molecular mechanisms and pathways of these clusters were defined through KEGG enrichment analysis. The results showed that the genes expressed by these three groups of cells could be enriched in NK-cell-mediated cytotoxicity and were associated with pathways such as autoimmune response and graft rejection (Fig. 3C). Thus, these three clusters of cells could be used as target cell subsets for our further study of URSA.

The differences in transcriptional levels between URSA and normal pregnant women were analysed in these three groups of cells. Interestingly, the numbers of differentially expressed genes in these three groups that were significantly upregulated and downregulated were also higher than those in other NK-cell clusters (Fig. 3D). The associated differentially expressed genes were enriched into three groups, and the expression levels of these genes in Clusters 3, 4, and 1 were compared by heatmap (Fig. 3E). The first heatmap mainly enriched genes associated with NK-cell cytotoxicity, such as *NFKBIA*, *FOS*, *TNFAIP3*, and *REL*. The expression of this group of genes decreased in the order of cell Clusters 3, 4, and 1, but the NK-cell reactivity of URSA patients increased significantly. The second heatmap enriched NK-cell characteristic genes, including granule protein genes such as *GNLY*, *GZMB*, and *GZMA*, and KIR genes such as *LILRB1*, *KIR2DL3*, and *KIR2DL1*. Compared with the control, granule proteins were generally upregulated, while KIRs were downregulated, especially in the Cluster 4 stage. *KIR2DL3* and *KIR2DL1* transcriptional levels decreased significantly in URSA dNK cells. The third set of heatmaps enriched ECM-related genes, a group of genes that were significantly downregulated in URSA patients, in which collagen expression was significantly downregulated, such as *COL1A1* and *COL1A2*. Additionally, glycodelin (*PAEP*), which is an immunomodulator in pregnancy, also decreased significantly in URSA samples.

### Pseudotime analysis of dNK cells in URSA

**Fig. 4:**
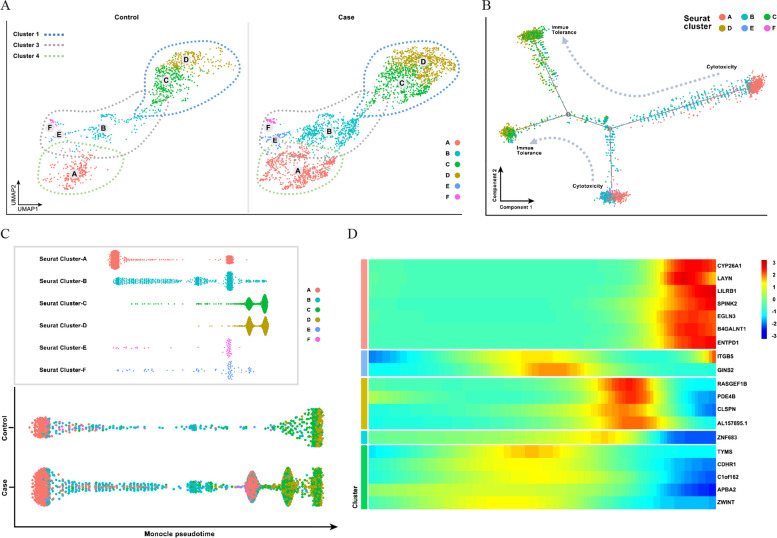
Pseudotime analysis of dNK cells in URSA. **A** Cluster 3 (A), 4 (B, E, F) and Cluster 1 (C, D) were further grouped. **B** Cluster 3 (A) and Cluster 1 (C, D) were located at both ends of the differentiation of dNK cells. **C** The dNK cell differentiation status of URSA patients and normal pregnant women. **D** The marker genes in the differentiation process of target dNK cells were obtained from the feature selection based on pseudotime analysis

To reveal the interrelationships between target NK cells, Clusters 3 (A), 4 (B, E, F) and 1 (C, D) were further grouped (Fig. 4A). We used Monocle 2 (http://cole-trapnell-lab.github.io/monocle-release/) to perform pseudotime trajectory analysis. The maturation of dNK cells is thought to be a transition from cytotoxicity to immune tolerance. In the pseudotime analysis, Cluster 3 (A) and Cluster 1 (C, D) were located at both ends of the differentiation of dNK cells. Cluster 1 (C, D) was considered to be mature dNK cells because of its immune tolerance, while Cluster 3 (A) was immature dNK cells with strong cytotoxicity, and Cluster 4 (B, E, F) was a stage of transition (Fig. 4B). The dNK cell differentiation status of URSA patients and normal pregnant women was compared in a single pseudotime analysis. The analysis results showed that the differentiation of dNK cells in URSA patients was disordered, and Clusters A, B, E, and F all stagnated during the differentiation stage, resulting in insufficient polarization of Clusters C and D, which should be mature (Fig. 4C). Simultaneously, the marker genes in the differentiation process of target dNK cells were obtained from feature selection based on pseudotime analysis, such as *CYP26A1*, *LILRB1*, and *B4GALNT1* in the polarization stage and *C1orf162*, *RASGEF1B*, and *ZNF683* in the transition stage (Fig. 4D).

### Characteristics of abnormally differentiated dNK cells in URSA

**Fig. 5:**
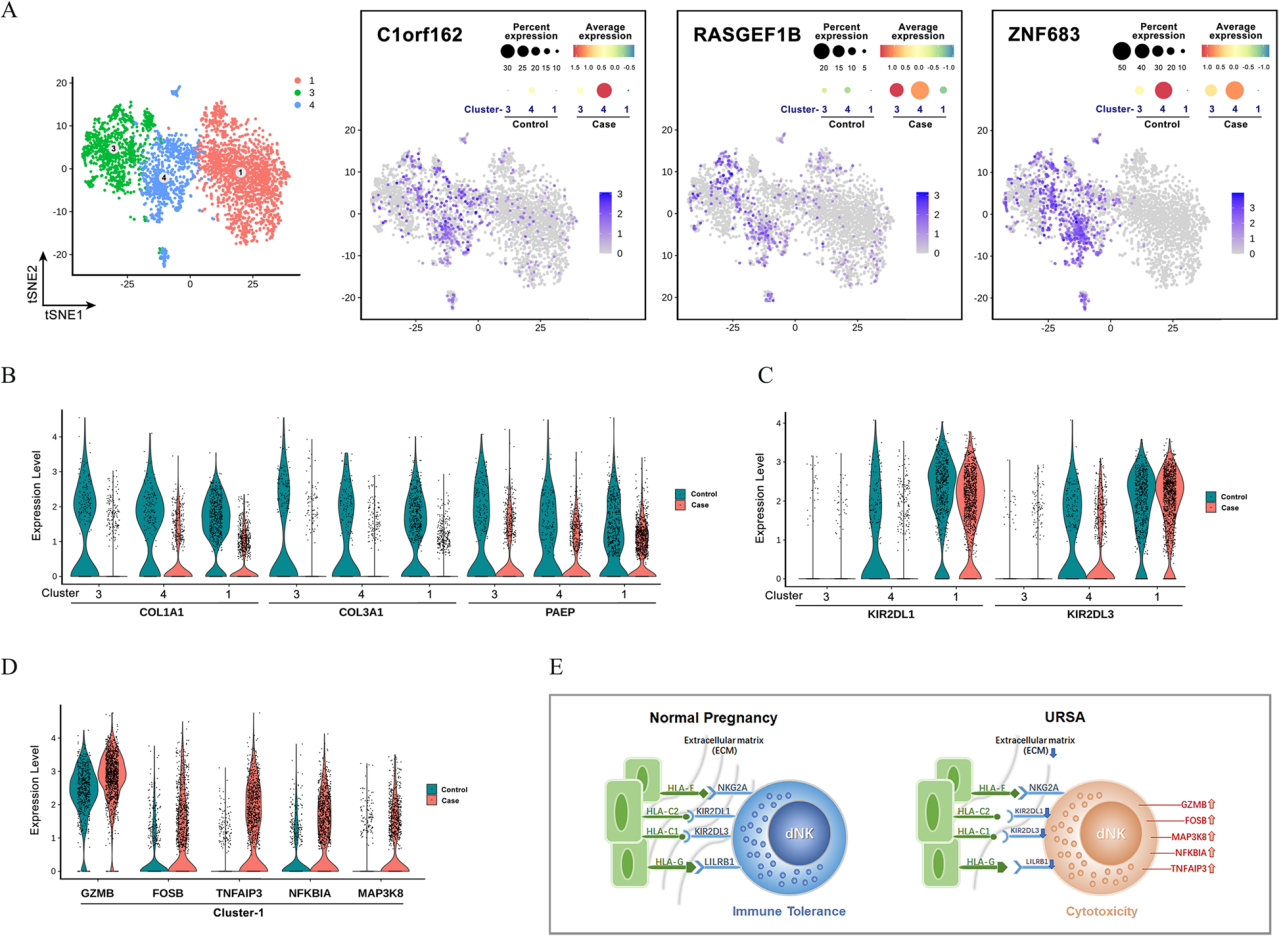
Characteristics of abnormally differentiated dNK cells in URSA. **A** Cluster 4 reflected the degree of disordered differentiation of dNK cells. **B** ECM was an indispensable factor for dNK cell maturation. **C** Decreased expression levels of *KIR2DL1* and *KIR2DL3* in Cluster 4 directly affected the state of mature dNK cells. **D** The mature dNK cells in URSA still had high cytotoxicity and immunoreactivity. **E** Diagram of the inadequate interaction of dNK cells with EVTs due to ECM deficiency

In this study, polarization disorders of dNK cells could be the key mechanism leading to URSA, where the phenotypic changes in intermediate cells, Cluster 4, directly reflected the degree of disorder of differentiation of dNK cells. *C1orf162*, *RASGEF1B*, and *ZNF683* could be used as Cluster 4 markers by tSNE clustering and difference analysis, while *C1orf162* and *RASGEF1B* would be of value for the diagnosis of URSA (Fig. 5A). ECM was an indispensable factor for dNK cell maturation, and the low expression of *COL1A1*, *COL3A1*, and *PAEP* at the maternal–fetal interface led to dNK cell differentiation disorders (Fig. 5B). Decreased expression of *KIR2DL1* and *KIR2DL3* in Cluster 4 directly affected the state of mature dNK cells (Fig. 5C), and even though KIRs could eventually be upregulated to normal expression levels, the mature dNK cells in URSA still had high cytotoxicity and immunoreactivity (Fig. 5D). All these pathological effects might stem from inadequate interaction of dNK cells with EVTs due to ECM deficiency (Fig. 5E).

## Discussion

Decidualization can be considered as a biological solution for adapting to maternal–fetal genetic differences during human evolution, and the invasiveness of embryos is designed to ensure maximum expansion of offspring genes [A], while also ensuring a high-quality fetal adaptive response during a longer pregnancy [B]. The decidualization process involves multiple immune cell types, transcriptional regulation, and cytokine interactions, while immune dysregulation at the maternal–fetal interface can trigger pregnancy failure. Research evidence suggests that IL-2, IFN-γ, TNF-α, IL-6, IL-8, IL-17, and IL-23 increase in URSA decidual tissue, whereas IL-4, IL-10, IL-22, IL-27, TGF-β, LIF, and MIF were reduced, and the shift in cytokines balance indicated a maintenance disorder of immune tolerance [C].

Imbalances in maternal–fetal interface immune tolerance are thought to be the main cause of URSA with immune disorder. NK cells are the main immune cells in the decidua, and in our analysis results, the proportions of NK and T cells in the decidua of URSA patients increased, and the increase in NK cells was more obvious. By comparing the differences between the cell-type composition and the immune cell transcriptome in the decidua of URSA patients and normal pregnant women by scRNA-seq, it was possible to comprehensively and systematically identify the features of immune dysregulation in URSA. Since the interaction between NK cells and EVTs formed the core link in the establishment of immune tolerance at the maternal–fetal interface, we focused on the changing characteristics of dNK cells in URSA patients through bioinformatics analysis.

In a successful pregnancy, different from peripheral blood CD16^+^CD56^dim^ NK cells, dNK cells typically have a CD16^−^CD56^bright^ surface phenotype with limited cytotoxicity [13, 21]. The acquisition of immune tolerance function by dNK cells is a process of maturation and differentiation that requires adequate interaction with EVTs [5, 7]. In our study, the three clusters of interrelated NK-cell subsets were annotated in UMAP clustering, and they were speculated to be 3 characteristic differentiation stages during the process of immune tolerance acquisition through pseudotime analysis. Interestingly, the results of our analysis were very similar to the three subsets of dNK cells identified by Tormo et al. [18]. The analysis showed that Cluster 1 cells, with *ENTPD1*, *CYP26A1*, and *B4GALNT1* as marker genes, highly expressed KIRs, including *KIR2D1*, *KIR2D3*, *KIR3D1*, and *LILRB1*, and contained more cytoplasmic granules than the other two groups of NK cells, including *GNLY*, *GZMA*, *GZMB* and *GZMK*. Therefore, Cluster 1 cells were deemed to be mature dNK cells that interact with EVTs, and this type of dNK cell maintained the balance between immune tolerance and immune effects during pregnancy. In the analysis of differences with normal pregnant women, the immune tolerance characteristics of cluster-1 cells in URSA patients decreased, while the propensity of cytotoxicity increased significantly. In addition to cytoplasmic granules, the levels of *FOSB*, *TNFAIP3*, *NFKBIA* and *MAP3K8* were enhanced. The imbalance of immune regulation might be a direct cause of miscarriage. In contrast to the immune tolerance of Cluster 1 cells, Cluster 3 cells with *ANXA1* and *ITGB2* marker genes had a pronounced propensity for cytotoxicity and expressed the lowest levels of KIRs among the 3 groups of dNK cells. The Cluster 3 cells might be the initial NK cells that had not yet interacted with EVTs and did not yet have property of immune tolerance. Finally, Cluster 4 NK cells, which were distributed between Cluster 3 and Cluster 1, were marked by *C1orf162*, *RASGEF1B*, and *ZNF683* and might be the essential differentiation process for the transfer of dNK cells to immune tolerance. It is worth noting that the transcriptional level of KIRs in URSA patients at the Cluster 4 stage was significantly downregulated, among which *KIR2DL1* and *KIR2DL3* differed significantly from the expression levels in normal pregnant women. Insufficient expression of KIRs during dNK cell differentiation might be a key reason why the polarized dNK cells still had high cytotoxic reactivity in URSA patients. The activation and cytotoxicity of dNK cells are affected by a variety of factors such as the cytokines environment and post-transcriptional regulation. Studies have focused on the role of miRNAs in pregnancy, in which miR-30e was found to be expressed in both peripheral blood and decidual tissues in RSA patients, while its downstream target gene PRF1 (Perforin 1) was upregulated, suggesting that miR-30e may be associated with the activation of dNK cells by negatively regulating PRF1. In addition, the miR-30e mimetic has also been shown to upregulate KIR2DL1 and downregulate the expression of the activated receptor NKp44, thereby regulating cytotoxicity in dNK cells [D].

In Monocle pseudotime analysis, we found that 3 clusters of dNK cells in normal pregnant women showed a pronounced polarized distribution, which suggested the process of smooth immune tolerance transfer. However, the NK cells in URSA patients revealed an inadequate and abnormal differentiation process. Many possible mechanisms lead to imbalances in the immune regulation of dNK cells during pregnancy. In our study, the expression of ECM genes decreased significantly in URSA patients, of which the downregulations of *COL1A1* (collagen type I) and *COL3A1* (collagen type III) were the most obvious. During pregnancy, the endometrium undergoes a series of changes, including collagen breakdown and remodelling. Collagen induces the expression of *KIR2DL1* in dNK cells [22]and inhibits STAT1 and STAT4 signalling through LAIR-1, reducing the cytotoxic effects of dNK cells [23]. It also plays a critical role in the immune tolerance of dNK cells. Additionally, glycodelin (*PAEP*) was reported to have immunosuppressive properties, which are indispensable for the maintenance of pregnancy [24]. In our analysis, the *PAEP* transcriptional level was significantly downregulated during dNK cell differentiation in URSA patients. Therefore, abnormal expression of ECM may affect the interaction of dNK cells with EVTs, making dNK cells immature and resulting in maternal immune intolerance to the foetus during pregnancy. However, the causes of URSA decidual immune tolerance disorders are diverse, such as insufficient progesterone secretion, which can lead to decreased expression of *COL1A1* and *COL3A1*. Therefore, the clinical treatment of URSA should still consider the specific condition of the patient.

## Methods

### Clinical samples

The maternal–fetal interface tissues were collected after obtaining written informed consent from 3 patients diagnosed with URSA and 3 normal pregnant women at Shuguang Hospital of Shanghai University of Traditional Chinese Medicine. This study has passed the ethical review (Ethics number: 2020–860-69–01). Certified pathologists with extensive experience performed the surgical resections of endometrial tissue for patients in this study.

### Single-cell solution preparation

The maternal–fetal interface tissues were cut into approximately 1–2 mm^3^ pieces and digested in digestion solution with type I collagenase, neutral protease and DNase 1 at 37 °C for 15–30 min. Then, the enzymatic digestion was stopped with excess RPMI-1640 medium, and the cells were filtered with a 40-μm cell strainer. Single-cell solution samples were kept on ice before loading to a BD Rhapsody™ cartridge for single-cell transcriptome capture.

### Single-cell transcriptome capturation, library construction and sequencing

Cells were first stained with two fluorescent dyes, Calcein-AM and DRAQ7, for precise determination of cell concentration and viability via a BD Rhapsody™ Scanner (BD Biosciences). Cells were loaded in one BD Rhapsody™ microwell cartridge based on Fan et al. [25]. Cell capture beads were then loaded excessively to ensure that nearly every microwell contained one bead, and the excess beads were washed away from the cartridge. After lysing the cells with lysis buffer, the cell capture beads were retrieved and washed prior to performing reverse transcription. The microbead-captured single-cell transcriptome was generated into a cDNA library containing cell labels and unique molecular identifier (UMI) information. All procedures were performed with a BD Rhapsody cDNA Kit (BD Biosciences, Cat. No. 633773) and a BD Rhapsody Targeted mRNA & AbSeq Amplification Kit (BD Biosciences, Cat No. 633801) strictly following the manufacturers’ protocol. All the libraries were sequenced in PE150 mode (paired-end, 150-bp reads) on the NovaSeq platform (Illumina).

### Sequencing data processing

Raw sequencing reads of the cDNA library were processed through the BD Rhapsody Whole Transcriptome Assay Analysis Pipeline (v1.8), which included filtering by read quality, annotating reads, annotating molecules, determining putative cells and generating a single-cell expression matrix. Among all output files, the matrix of UMI counts for each gene per cell was used for downstream analysis. Genome Reference Consortium Human Build 38 (GRCh38) was used as a reference for the BD pipeline.

### Dimensionality reduction, clustering and visualization

Seurat was used for subsequent clustering analysis and visualization [26, 27]. Gene expression matrices for each sample were read and converted to Seurat objects. Cells with more than 25% mitochondrial UMI, less than 500 UMI or 200 genes were excluded from the downstream analysis. After log normalization according to the total cellular UMI count, PCA was performed based on the top 2000 highly variable features after scaling the data with respect to UMI counts. Then, we performed clustering at a resolution of 0.6 and visualized the data using either t-distributed stochastic neighbour embedding (t-SNE) or uniform manifold approximation and projection (UMAP). Feature plots, violin plots and heatmaps were used to visualize the expression of the indicated genes in each cluster**.**

### Differentially expressed gene analysis

To identify specific markers between different identities, we used Find-Markers (Seurat R Package) with the Wilcoxon Test under the following criteria: min. pct > 0.25. Genes were regarded as upregulated when their log2 fold change > 0.25 and *p* value adjustment < 0.05, and genes were regarded as downregulated when their log2 fold change < -0.25 and *p* value adjustment < 0.05.

### Cell type annotation

Specific markers were calculated for each cluster using the Find-All-Markers function with the Wilcoxon Test under the following criteria: log2 fold change > 0.25; min. pct > 0.25. To unbiasedly identify cell type in filtered sample datasets and the combined dataset, we used the R package SingleR (v1.4.1) [28], a computational framework by reference to bulk transcriptomes helping us annotate the cell types for each cluster.

### Pseudotime trajectory analysis

After cell type annotation, single-cell pseudotime analysis was performed for each cell type separately using Monocle 2 with the DDR-Tree reduction method [29]. Single-cell pseudotime analysis was performed with default parameters. Briefly, a Monocle object was first created according to the expression matrix and metadata information stored in the Seurat object. During feature selection, the top marker genes of Seurat clusters were set as the ordering genes for downstream analysis. Batch effects were also eliminated during dimensionality reduction. Trajectory plots and heatmaps were used to show the pseudotime results.

## Supplementary Information

**Additional file 1:** **Supplement Table 1. **A total of nearly 30000 marker genes were identified in all cell samples.

**Additional file 2:** **Supplement Table 2. **Marker genes significantly expressed by each cell cluster.

## Data Availability

The datasets used and/or analysed during the current study are available in the additional files.

## Abbreviations

URSA: Unexplained recurrent spontaneous abortion
dNK: Decidual natural killer
scRNA-seq: Single-cell RNA sequencing
ECM: Extracellular matrix
EVTs: Extravillous trophoblasts
MHC-I: Major histocompatibility complex class I
KIRs: Killer immunoglobulin-like receptors
UMAP: Uniform manifold approval and projection
DCs: Dendritic cells
VCTs: Villous cytotrophoblasts
